# The Basic Cycles of Vocational Training: Student Satisfaction and Perceived Benefit

**DOI:** 10.3390/ejihpe12040030

**Published:** 2022-04-01

**Authors:** María José Martínez-Carmona, Carmen Gil del Pino, José Luis Álvarez-Castillo

**Affiliations:** Department of Education, Faculty of Education Sciences, University of Córdoba, 14071 Cordova, Spain; ed1gipim@uco.es (C.G.d.P.); ed1alcaj@uco.es (J.L.Á.-C.)

**Keywords:** transition decisions, secondary school, school tracks, revealed students’ preferences, satisfaction, quantitative method, optimization

## Abstract

This study aims to better understand students who attend Basic Vocational Training Cycles (Basic Professional Training, BTP) by implementing measures that ensure diversity. This quantitative research project approximated students’ perception of their passage through previous studies and their satisfaction and goals after finishing the school year. The sample consisted of 352 students from Cordoba (Spain). A questionnaire was used that follows the CIPP model. After exploratory factor analysis was completed with different groups of items and their descriptive analyses, various tests were carried out to consider the hypotheses (Pearson’s correlation (*r*), one-factor analysis of variance, and repeated ANOVA measures). The results indicate that the educational interest of the students is academic and professional. Likewise, there is no relationship detected between socio-professional goals and average academic levels and attributions with respect to repetitions of previous courses, although these goals vary depending on students’ satisfaction with the vocational cycles. We conclude that the course of the FPB influences decisions regarding academic–professional projects.

## 1. Introduction

Permanent education is a present need of the population [1,2]. The global market generates a degree of competitiveness and uncertainty characterized by the constant change in regulations worldwide. The objective is to adapt training to the market and companies while also covering new sources of employment [3]. On the other hand, it is necessary to promote civic–social skills with methodological strategies and adjusted programs aimed at the transition to employment [4]. With this in mind, attractive, innovative, and dynamic Vocational Education and Training (VET) is considered [5,6,7,8,9].

The current vision defended by various national and world bodies is that VET (Vocational Education and Training) is the method best adapted to the reality of the labor market and the needs of the world economic system. The aim is to provide qualified and specialized personnel to productive professional sectors and to satisfy the demand for employment [10,11,12]. In this sense, a wide catalog of Training Cycles is made possible within various professional fields [9], each specified with theoretical–practical content that helps future workers develop specialized skills. Depending on the level of study required, these cycles are divided into Basic Professional Training (BTP, in Spain, FPB), Middle-Grade, and Higher-Grade Vocational Training Cycles.

The Basic Cycles, the object of this study, grant the Basic Professional Title, and in Spain, they are offered on a compulsory and free basis. In order to promote them, the goal is to generate a culture of innovation and risk-taking [6] that encompasses all scales of the production system and society, especially in education and training. One of the European objectives is innovation, the axis of modernization of VET [13,14,15,16].

Organic Law 8/2013, of December 9, *para la mejora de la calidad educativa* (LOMCE), outlines how the rigidities of the system can be exclusive to a part of the student body because they have different interests from those set by the system itself [17,18,19,20]. Letting students follow different paths can help reduce school dropouts and improve personal and professional development. Basic Professional Training (BPT) is one possible option [21]. Historiographic synthesis studies [22] analyze the expression of professional training and the variability of its interpretation, as well as its correlation and subordination to lifelong learning, according to a historical and comparative or international axis. From a technical–analytical approach to standardized mass production—typical of Taylorism and considered in the 1990s to be a sign of progress and growth [23], in which the ideal worker endured monotony and hard work—to the study of Lean production [24], in which the worker has learned to adapt to the needs of production, education has played a fundamental role in adaptation. This eminently socializing character of the school drives the modernization of the search for a model with less youth unemployment [25,26]. We evoke Durkheim [27] in recalling that the purpose of education is to elicit physical, intellectual, and moral states demanded by political society—as a whole—and the specific environment it is specifically destined for.

For a student to be admitted to BPT, they must meet the following requirements simultaneously: be between fifteen and seventeen years old, have started Compulsory Secondary Education (ESO), and be proposed by the teaching team for incorporation into a BPT cycle [19]. The program’s usual duration is two years.

Upon successful completion of the professional modules, the student obtains a Level One qualification from the National Catalog of Professional Qualifications [25] and the title of Basic Professional, which grants access to the Middle Degree Training Cycles, although it does not grant the ESO title, which requires an additional knowledge assessment [17].

### BPT Student Profile

The United Nations Development Program [28] considers young people’s vulnerability to marginalization in the labor market due to unemployment, underemployment, or precarious contracts [29]. Likewise, transitions during youth become uncertain, and the de-standardization of normalized before–after models—of a more reliable and predictable nature—reveals the end of the linear cause–effect relationship [29,30,31,32,33,34]. The EGRIS (European Group for Integrated Social Research) network assessed the unwanted effects of social exclusion and labor market policies on young people in Europe [35], with the participation of research teams from Denmark, Germany, Ireland, Italy, the Netherlands, Portugal, Spain, and the United Kingdom. Research on youth and the labor market shows that many young people choose, or are forced to choose, educational options that do not lead to stable jobs or socially accepted status; others drop out or completely withdraw from the system, preferring a zero status in which they experience alienation and humiliation [30]. Various authors show us the possibility for youths to escape these closed circuits [8,35,36,37,38,39], discussing the need for positive experiences, the positive development of personal identity [40,41], and the meaning of work as elements for optimization and issues of vital importance for BPT [42,43,44].

In this regard, the research indicates that the current measures seeking diversity could lead to the exclusion of students rather than inclusion [45,46,47]. Other studies [48,49] reveal little collaboration between the teaching staff, the counselor, and other members of the educational community in the implementation of the cycles; their short duration results in the selection of incomprehensible content and objectives. Likewise, planning is usually short-term and adapted to the student’s needs, and the student must renounce interest in studies [50] because, after the student’s failure in the traditional system, the cycles cannot offer more of the same [48,51,52]. Finally, taking into account that many of the students in the BPT program were unsuccessfully guided through Secondary Education, orientation and tutoring should be promoted in BPT so that students can set goals and expectations [52,53] according to the peculiarities of the context and the students themselves [54,55].

## 2. Materials and Methods

In a broad sense, this research was based on rigorous and objective analysis of the educational situation [55,56,57]. We intended to learn about the educational reality of students in BPT. We highlighted the following objectives: to learn about student experiences and perceptions concerning the courses not passed, show their satisfaction with BPT, and learn about their expectations after completing their studies. The research design was flexible and adapted to the context. From the rationalist paradigm and a quantitative perspective, this study is defined as descriptive, applied, and evaluative.

### 2.1. Participants

The sample was drawn from 27 Secondary Education centers in Cordoba (Spain) out of the 60 that offer BPT, with 352 students surveyed during the 2016–2017 academic year.

In regard to personal and context data, the students were between 14 and 19 years old. A total of 72.2% were men, and 27.8% were women, a percentage comparable to that recorded in MECD and MEFP reports [14,15]. The study covered the following specialties: office computing (*n* = 27), administrative services (*n* = 46), agro-gardening and floral compositions (*n* = 54), electricity and electronics (*n* = 59), kitchen and catering (*n* = 19), hairdressing and aesthetics (*n* = 15), communications (*n* = 81), carpentry and furniture (*n* = 17), domestic activities and building cleaning (*n* = 8), vehicle maintenance (*n* = 19), and food industries (*n* = 7). There are specialties for which a smaller sample has been obtained because there are few centers that offer studies, while others are taught in a large number of centers.

The reasons why the students studied a cycle were: “To have a job” (74.5%, *n* = 254); “that they feel proud of them” (65.1%; *n* = 222); “be prepared for their professional future” (50.6%, *n* = 171). On the other hand, 50.6% of the students repeated in Primary and 92.4% in Secondary (49% repeated once and 43.5% twice). These data indicate a history of failure throughout the students’ school life.

### 2.2. Instruments

This quantitative research has been carried out through statistics. A comprehensive model that combines the perspective by phases and areas with the global perspective was used to evaluate the process and observe how the educational system (in general) and educational centers (in particular) reach their objectives.

The instrument used was a questionnaire. Due to the limited existing studies in this area, it was necessary to complete exploratory factor analyses with the groups of items that make up this tool, structured in blocks through the CIPP model (Stufflebeam), with the following scheme: context or input (information related to the description of the sample and reasons why the students chose to study a cycle, number of times they have repeated and school stage), process (type of activities carried out during the course of the cycle, their assiduity and degree of satisfaction of the students), and product (expectations of students at the end of the cycle and goals). Open, closed, and Likert-type questions were alternated with the possibility of answers ranging from 1 to 5, adding the option “I don’t know/I don’t answer” where appropriate.

The process of preparing the questionnaire has been constituted in various stages, the last of which consisted of review by experts belonging to this scientific specialty. After informing about the purposes and objectives of this research, the validation of the questionnaire consisted of scoring the values of clarity, relevance, contribution of a possible alternative format, and comments on the matter. A table was provided to indicate whether the values for the items in the questionnaire and as a whole were: insufficient or unacceptable; sufficient or acceptable; very sufficient or fair (on a three-grade scale). Information was also requested regarding the relevance of the questions in relation to the objectives, their wording, suggestions and recommendations.

### 2.3. Procedure

In the first place, participation in the study was requested from the Management Team and/or the Guidance Department of the chosen centers, with a fairly satisfactory reception. For the first contact, a cover letter was used accompanied by a summary of the project, its purpose and contact information. After obtaining the approval of the different centers, we held some informal interviews with directors, counselors and teachers, showing interest in the study and its relevance.

Next, the periods for applying the questionnaires were established, guaranteeing the anonymity and confidentiality of the data collected. This study complied with the Declaration of Helsinki and with Andalusia (Spain) guidelines on research with human subjects and protection of privacy (Decree 8/2020, of January 30). No sensitive data that could identify the participants were collected. The required institution gave their consent and approval to participate in the study of underage students (20 June 2016). The study followed the *Code of Responsible Practices and Integrity in Research* of the University of Córdoba (BOUCO 12/19/2015).

### 2.4. Analysis

The validity of the construct was determined using exploratory factor analysis through the SPSS statistical program. To this end, the Bartlett sphericity test was applied to each group of items, which made it possible to continue with the extraction of factors. The Kaiser–Meyer–Olkin sample adequacy measure was used to determine the acceptable degree of common variance between the items. The solution was initially rotated with Varimax, but the factors proved reliable and correlated significantly and with some intensity. The initial decision was reconsidered, and an oblique rotation method was adopted (Oblimin with Kaiser normalization (delta = 0)). The reliability of the survey and the various resulting factors were found using Cronbach’s Alpha internal consistency analysis.

After the interpretation of reliable factors and the high correlation between them, the functioning of the variables was evaluated descriptively, and we proceeded to contrast the hypotheses raised with the predictive values through the Pearson correlation test (*r*), one-way ANOVA, and repeated ANOVA measures based on their normal distribution and the type of variables analyzed in each case. For the latter analyses, the Kolmogorov–Smirnov statistical test was not chosen to determine if the data fit a normal distribution because ANOVA is considered a robust test by itself. The assumption of homoscedasticity was previously verified in the Levene test.

## 3. Results

The results shown below are divided into blocks: school records, attributions regarding repetitions of previous courses, current educational interest, satisfaction with respect to BPT cycles, and goals after completing the cycle.

### 3.1. School Records

Descriptive data regarding the repetitions of students in previous courses are shown below (Table 1).

About half of the students repeated courses in Primary Education (*N* = 352), a figure that increases in Secondary Education (92.4%, *n* = 316), in which 49% repeated courses once and 43.5% repeated courses twice. The failure of these students in school is evident, and it is essential that we review the students’ attributions in this regard, as detailed below.

### 3.2. Attributions Regarding Repetitions of Previous Courses

An exploratory factor analysis was carried out using principal component analysis. Bartlett’s sphericity test showed a significant difference between the empirical correlation matrix and the identity matrix ($x^{2}$ [231] = 1400.08, *p* < 0.001), which made it possible to continue with factor extraction. Likewise, the Kaiser–Meyer–Olkin measure of sampling adequacy reported an adequate degree of common variance between the items (KMO = 0.801).

The criterion of an eigenvalue greater than one was maintained since it yields a solution of six fairly uniform factors in terms of explained variance, from which the adequacy of the model was deduced to explain the correlational matrix. The variance explained by the six-dimensional solution was estimated at 55.89%, sufficient for subsequent analyses.

The solution was initially rotated with Varimax, but the resulting factor score distributions in the factors that were shown to be reliable significantly correlated with some intensity, which led to the rethinking of the initial decision and the adoption of an oblique rotation method (Oblimin with Kaiser normalization (delta = 0)). The rotated model is presented in Table 2, which shows the saturations of the items in the factors.

The regression method was used to obtain an estimate of the factorial scores, verifying a high correlation between factors 1 and 2 (*r* = 0.510, *p* < 0.001); the correlation was moderate between factors 1 and 6 (*r* = 0.429, *p* < 0.001), 3 and 5 (*r* = 0.352, *p* < 0.001), 2 and 6 (*r* = 0.342, *p* < 0.001), 4 and 5 (*r* = 0.321, *p* < 0.001); and light among factors 2 and 4 (*r* = 0.296, *p* < 0.001), 2 and 3 (*r* = 0.274, *p* < 0.001), 2 and 5 (*r* = 0.216, *p* < 0.001), 3 and 4 (*r* = 0.184, *p* = 0.001), 1 and 5 (*r* = 0.159, *p* = 0.003), and 1 and 4 (*r* = 0.154, *p* = 0.005).

Based on the content of the items that are most significant in each factor, the following interpretation was reached:

(a) Factor 1: *Lack of integration and non−improvement* (IS). This category refers to the lack of integration of the student into the class group, discrimination and rejection (47, 55, and 57), and the belief that they will not successfully complete ESO (53, 63, and 65).
(b) Factor 2: *Externalization of failure* (EF). These items (52, 54, 56, 59, and 60) refer to external causes for being held back, such as the lack of usefulness of the lessons, underestimation of the student’s effort, or the teachers’ lack of interest.
(c) Factor 3: *Multiplicity of resources making learning difficult* (MR). This category considers the large number of teachers and subjects to study during ESO and the difficulty in following explanations and classes (44, 45, and 46).
(d) Factor 4: *Disruptive behavior and indifference* (DB). This category groups the consequences of inappropriate behaviors in the classroom and apathy towards studies (58, 61, and 64).
(e) Factor 5: *Internalization of failure* (IF). This category alludes to a lack of effort and repeater labeling (49, 50, and 51), showing intrinsic causes.
(f) Factor 6: *Change of center without support* (CA). This category refers to a lack of family support and the search for improvement through a change of educational center (48 and 62).

Once the construct structure was explained, the internal consistency was calculated. The reliability coefficients in the six factors (Cronbach’s Alpha) were 0.735, 0.714, 0.603, 0.556, 0.505, and 0.404, respectively. The internal consistency of the construct was adequate in the first two factors, so its use was maintained for critical analyses, although successive factors were rejected.

Considering the reliability of the factors and the high correlation between them, which show the existence and association of several factors used to explain repetition during ESO, it was decided to use the following two dependent variables in later analyses: *lack of integration and non−improvement* (IS) and *externalization of failure* (EF).

The results describing each of the latent variables are presented below. The following table (Table 3) shows that the students did not agree much regarding a lack of integration and failure to pass (IS), with a mean of 2.18 (*N* = 341) and a cumulative response percentage of 56.7%. The students neither felt discrimination or rejection from other classmates nor felt different from the group. They did not believe that the teachers “gifted” them passing grades. Likewise, students showed little agreement regarding the externalization of failure (EF), with a total mean of 2.60 (*N* = 342, 53.8%). This indicates that students believed that the teaching staff was interested in becoming acquainted. Even though they failed, this effort was valued.

### 3.3. Current Educational Interest

In order to find the benefit of BPT for students, the following variables were considered: interest in academic–professional training, only professional interest, only academic interest, and a lack of interest. To analyze the differences, repeated ANOVA measures were completed with the four intra−subject variables (Table 4). Significant differences affecting the sample group were found for all current educational interest variables (*F* [3, 945] = 151.95, *p* < 0.0001, ε2 = 0.325).

**Figure 1:**
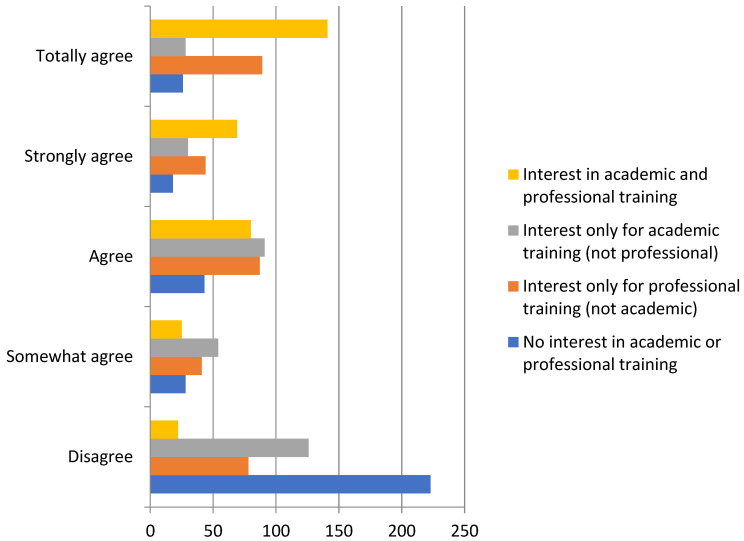
Interest in academic and professional training.

The union of academic and professional training was valued by almost half of the students surveyed (41.8%, *n* = 141), with an average response of 3.85 (*n* = 316). As shown in Figure 1, the students did not agree that they were only interested in academic training (*n* = 126, 38.3%), with professional training being the most valued. The lowest mean of response referred to a lack of interest (M = 1.81).

### 3.4. Satisfaction with Respect to BPT

An exploratory factor analysis was carried out using principal component analysis. Bartlett’s sphericity test showed a significant difference between the empirical correlation matrix and the identity matrix ($x^{2}$ [153] = 2197.633, *p* < 0.001), making it possible to continue with factor extraction. Moreover, the Kaiser–Meyer–Olkin measure of sampling adequacy reported an adequate degree of common variance between the items (KMO = 0.925).

The extraction of factors was forced to two since this yields a more uniform and adequate solution to explain the correlational matrix. The variance explained by the three−dimensional solution was 49.32%, acceptable for subsequent analyses. As in the previous exploratory factor analysis, an oblique rotation method was adopted (Oblimin with Kaiser normalization (delta = 0)), as shown in Table 5. The regression method was used to estimate the factor scores, verifying high correlations between factors 1 and 2 (*r* = −0.614, *p* < 0.001).

Based on the content of the items that are most significant in each factor, the following interpretation was reached:

(a) Factor 1: *External support and adaptation to the cycle* (AP). This category refers to the help and the proper number of teaching staff (99, 98, 97, 106, and 95), help from classmates (105) and families (109), and the suitability of the cycle (100, 107, 96, and 108).
(b) Factor 2: *Empowerment and improvement of the situation* (EE). This category highlights the student’s positive attitude and enthusiasm for learning (104, 103, 101, 110, and 111) and growth compared to previous studies (102, 94).

Once the construct structure was explained, the internal consistency was calculated. The reliability coefficients (Cronbach’s Alpha) were 0.876 and 0.848, respectively. The internal consistency of the construct was very strong in both factors, so its use was maintained for critical analyses. When considering the reliability of the factors and the correlation between them, it was decided to use two dependent variables in subsequent analyses: *external support and adaptation to the cycle* (AP) and *empowerment and improvement of the situation* (EE).

After the descriptive analysis (Table 6), a high degree of agreement could be observed regarding the satisfaction given to external support and the adequacy of the cycle (M = 3.90, *N* = 335). Likewise, the group agreed with the value placed on empowerment and the improvement of the situation (M = 4.01, *N* = 335).

One hypothesis is that the specialty influences the students’ degree of satisfaction regarding development in the studies carried out. To verify this, we analyzed the variance of a factor (*n.s*. = 0.05).

The AP and EE variables were considered. Levene’s test for homogeneity of variances showed values greater than 0.05 in two variables: AP (*F* (3. 331) = 0.143, *p* = 0.934) and EE (*F* (3. 331) = 0.237, *p* = 0.870). As can be seen in Table 7, after the ANOVA measure of one factor, it is clear that there are significant differences in the scoring AP (*F* = 2.636; *p* = 0.050, **ε^2^** = 0.023) since there are no differences in EE by sectors (*F* = 1.032; *p* = 0.379, **ε^2^** = 0.009). Because a very specific difference was detected, we rejected the idea that the specialty influences the students’ degree of satisfaction in developing in their studies, which is very high in all sectors.

### 3.5. Goals after Finishing the BPT Cycle

An exploratory factor analysis was carried out. Item 120 (“I have not thought about it yet”) was eliminated when it was found that it did not contribute to the instrument’s reliability, which increased up to 0.762 using the remaining items. Bartlett’s sphericity test showed a significant difference between the empirical correlation matrix and the challenges or expectations after the BPT matrix ($x^{2}$ [28] = 585.50, *p* < 0.001), which made it possible to continue with factor extraction. Likewise, the Kaiser–Meyer–Olkin measure of sampling adequacy reported an adequate degree of common variance between the items (KMO = 0.790).

The criterion of an eigenvalue greater than one was used since it gave a solution of two similar factors. The variance explained by the two−dimensional solution was estimated at 54.14%, sufficient for subsequent analyses. The solution was rotated with Varimax. The rotated model is presented in Table 8, which shows the saturations of the items in the factors. The regression method was used to estimate the factorial scores, verifying a moderate correlation between the two factors (*r* = 0.371, *p* < 0.001); therefore, the adopted criterion was maintained.

Based on the content of the items that are most significant in each factor, the following interpretation was reached:

(a) Factor 1: *Socio−professional and intermediate academic levels goals* (MP). This category refers to short− and medium−term goals at a professional and academic level, basic and middle studies, and adds social purposes (112, 113, 114, 115, 117, and 119).
(b) Factor 2: *Long−term academic goals* (LP). This category converges the desire to go to university or higher education and continue studying long−term (116 and 118).

After reviewing the structure of the construct, the internal consistency was calculated. The reliability coefficients in the two factors (Cronbach’s Alpha) were 0.765 and 0.574, respectively. As only the first case was adequate, only the following variable is used in critical analyses: *socio−professional and intermediate academic levels goals* (MP).

In a descriptive analysis, it is clear that students highly value socio−professional goals and medium academic levels, giving higher value to obtaining an ESO Diploma (Table 9).

Next, we analyzed the hypothesis that the appearance of socio−professional goals and average academic levels is influenced by the students’ attributions about the repetition of previous courses. The MP variable and the IS and EF variables were taken into account. After performing Pearson correlation (*r*), no significance was detected at a level of 0.01 (bilateral) between MP and IS (*r* = 0.036, *p* = 0.515), nor with EF (*r* = 0.002, *p* = 0.976). These are very low or null relationships that cannot confirm the hypothesis raised. Considering both the lack of student integration and discrimination and rejection (IS), as well as the external causes due to which the student could have repeated prior grades (EF), the data indicated that students did not necessarily agree with these attributions (M = 2.18 and 2.60, respectively).

With the hypothesis that the socio−professional goals and average academic levels vary depending on the satisfaction shown by the students with respect to the BPT cycles, the MP variable, and the AP and EE variables were taken into account. Using Pearson’s correlation (*r*), significance was detected at the 0.01 level (bilateral), with a moderate relationship between MP and AP (*r* = 0.569, *p* < 0.001) and EE (*r* = 522, *p* = < 0.001). In this manner, we can indeed affirm that the goals that the students set after completing BPT are related to their satisfaction when taking these cycles.

## 4. Discussion and Conclusions

We rely on the results of research [20,33,36,37,38,39] since not all students without an ESO Diploma are the same, and their various itineraries are different from one another. The attributions that the students chose with respect to the repetition of previous courses were not linked to a lack of integration and failure to pass or to the externalization of failure.

On the other hand, the students value the academic–professional nature of the cycles and make us question whether these cycles really offer the opportunity to re−engage learning.

The UNDP [28] spoke of the vulnerability of young people without studies who could improve their social and professional development and their educational opportunities through BPT, as well as their perception, self−esteem, motivation, and responsibility, resulting in improvement in the level of inclusion in labor and educational contexts [43,47,50,52]. This possibility is affirmed. We concluded that the Basic Professional Training course influenced the adoption of decisions regarding academic–professional projects. Prior studies pointed to such a relationship [43]. More than a few authors have spoken about turning BPT into a measure capable of intervening in the socialization and work identity of students [33,34,52,54].

## 5. Conclusions

We are committed to an inclusive education based on welcoming students who do not wish to enroll in a closed and inflexible system. This research results from the decisions adopted in educational policy, which leads to reflection. We recommend a flexible, inclusive BPT, guaranteeing equal opportunities. It would be interesting to inquire about how BPT and secondary school teachers conceive of inclusion.

The main objective of the reforms should be to facilitate students’ educational success defined by inclusion and equity. Creating itineraries early on can generate school objectors, failure, and school dropouts. An educational system that allows students to disengage maintains and generates social inequalities and promotes precarious jobs and social exclusion. As an alternative, we propose that this question continue to be investigated, seeking less−devastating alternatives than those of previous policies, striving for solutions that are transdisciplinary, eco−forming, and sustainable.

Perhaps it would be convenient for these cycles to find room for intervention and to provide young people with the appropriate resources when and how they need them to handle the deprivation, vulnerability, and exclusion they are going through, as opposed to other forms of integration that stop and correct their deterioration trajectories. This may be an appropriate response capable of affecting young people’s socialization and work identity, as proposed by various authors. We propose following this proposal and generating research regarding social inclusion.

The latest educational laws being implemented are modifying the BPT, providing an uncertain future. In the face of such continuous changes, it is appropriate to propose a paradigmatic and non−programmatic change in thinking to confront the education of the future and the increasingly broad, deep, and serious inadequacy that lies between our knowledge and the increasingly poly−disciplinary, multidimensional, transnational, global, and planetary realities.

## Figures and Tables

**Table 1:** Repetitions of BPT (Basic Professional Training) students in Primary and Secondary Education.

Repetitions in Primary Education				Repetitions in Secondary Education				Nº Repetitions in Secondary Education					
YES		NO		YES		NO		1		2		>2	
*n*	%	*n*	%	*n*	%	*n*	%	*n*	%	*n*	%	*n*	%
171	50.6	167	49.4	316	92.4	26	7.6	144	49	128	43.5	18	6.1

**Table 2:** Rotated configuration matrix. Attributions regarding repetitions.

Items	Factors					
	1	2	3	4	5	6
47. I was not integrated into the new class, it was not “my group”	**0.754**	0.007	0.126	−0.030	0.008	0.121
57. I felt rejection from classmates	**0.743**	0.250	−0.089	−0.082	−0.030	0.070
55. I felt unfairly discriminated against	**0.707**	0.191	0.034	−0.088	−0.048	0.230
53. They “gifted me” some passing scores	**0.568**	0.083	0.084	0.175	−0.065	−0.113
65. I was not going to pass ESO for reasons beyond my control	**0.446**	0.253	−0.082	0.220	0.159	0.331
63. I was not going to pass ESO for personal reasons	**0.423**	0.124	−0.052	0.192	0.125	0.363
60. I liked having reinforcement in different subjects	0.127	**0.689**	−0.155	−0.100	0.210	−0.027
59. My effort was not valued	0.283	**0.674**	0.073	0.177	−0.003	0.090
56. They suspended me for no reason	0.214	**0.543**	0.194	0.226	−0.324	0.134
52. The teachers weren’t trying to get to know me	0.262	**0.542**	0.316	0.036	0.094	0.220
54. What I was studying was not useful	−0.012	**0.497**	0.371	0.083	0.066	0.327
46. There were too many study subjects during the same course	−0.086	0.235	**0.763**	−0.039	−0.076	0.058
45. There were too many teachers for the same course	0.203	0.101	**0.702**	0.037	0.229	−0.015
44. I did not follow the explanations and classes well	0.027	−0.191	**0.647**	0.189	0.274	−0.126
64. I was expelled from the center on some occasion	−0.009	0.017	−0.052	**0.785**	−0.040	0.128
61. I was punished on some occasion for no reason	0.075	0.472	0.089	**0.608**	0.085	−0.195
58. I was indifferent to the course. I did not care about anything	0.059	0.012	0.240	**0.605**	0.275	0.167
50. I didn’t try hard enough	−0.041	0.001	0.114	0.049	**0.817**	0.082
49. I was bored studying	−0.153	0.102	0.327	0.289	**0.604**	−0.107
51. I felt like “the repeater”	0.365	0.266	0.077	−0.027	**0.454**	0.058
62. I changed schools during my studies to improve	0.001	0.186	0.007	0.131	−0.048	**0.789**
48. I had no support from my family	0.321	−0.032	−0.008	−0.042	0.033	**0.606**
**Explained variance**	12.95	10.87	9.24	7.85	7.57	7.37

**Table 3:** Responses according to repetition in previous stages.

	M	DT	*N*
Factor 1: Lack of integration and non−improvement (IS)			
47. I was not integrated into the new class, it was not “my group”	2.39	1.49	337
55. I felt unfairly discriminated against	1.95	1.34	336
57. I felt rejection from classmates	2.06	1.42	338
53. They “gifted me” some passing scores	1.91	1.31	337
63. I was not going to pass ESO for personal reasons	2.31	1.45	337
65. I was not going to pass ESO for reasons beyond my control	2.42	1.50	334
Total:	2.18	0.93	341
Factor 2: Externalization of failure (EF)			
52. The teachers weren’t trying to get to know me	2.59	1.47	336
54. What I was studying was not useful	2.67	1.46	335
56. They suspended me for no reason	2.23	1.42	335
59. My effort was not valued	2.67	1.50	335
60. I liked having reinforcement in different subjects	2.85	1.48	327
Total:	2.60	1.00	342

**Table 4:** Intrasubject differences regarding educational interests.

(I) Factor 1	(J) Factor 1	Difference of Means (I–J)	Standard Error	Sig. ^b^
Academic and professional	Professional	0.766 *	0.122	0.000
	Academic	1.532 *	0.105	0.000
	Neither	2.044 *	0.109	0.000
Professional	Academic and professional	−0.766 *	0.122	0.000
	Academic	0.766 *	0.093	0.000
	Neither	1.278 *	0.096	0.000
Academic	Academic and professional	−1.532 *	0.105	0.000
	Professional	−0.766 *	0.093	0.000
	Neither	0.513 *	0.085	0.000
Neither	Academic and professional	−2.044 *	0.109	0.000
	Professional	−1.278 *	0.096	0.000
	Neither	−0.513 *	0.085	0.000

**^b^***p* < 0.05. * Adjustment for multiple comparisons: least significant difference (equivalent to no adjustment).

**Table 5:** Rotated configuration matrix. Student satisfaction.

Items	Factors	
	1	2
99. The teachers know me a lot, they care about me	**0.858**	0.205
100. The Cycle is more relaxed, it gives me time to learn	**0.669**	−0.123
98. I value having fewer teachers per course	**0.631**	0.102
108. Studying a Cycle improves my self−esteem	**0.622**	−0.188
97. The teachers facilitate my study. they are colleagues	**0.611**	−0.096
105. I have good classmates and that helps me	**0.560**	−0.110
107. Studying a Cycle is useful	**0.523**	−0.244
109. Studying a Cycle helps me with my family	**0.487**	−0.152
96. I feel like part of the class and they pay attention to me	**0.440**	−0.347
106. If I don’t understand something. they repeat it until I understand	**0.440**	−0.287
95. The teachers of the cycles help me	**0.381**	−0.378
102. I’m better in the Cycle than in the ESO	−0.176	**−0.887**
104. I come to class excited. it’s worth the effort	0.068	**−0.729**
94. This is the best thing that has happened to me in regard to my education	−0.007	**−0.728**
103. Now I am able to take on my own projects	0.102	**−0.703**
101. I come to class at ease. happy	0.162	**−0.656**
110. My attitude has improved	0.155	**−0.572**
111. I think I will pass the Cycle without problem	0.081	**−0.560**
**Explained variance**	42.93	6.38

**Table 6:** Student satisfaction with the BPT cycles.

	M	DT	*N*
Factor 1: External support and adaptation to the cycle (AP)			
99. The teachers know me a lot. they care about me	3.66	1.16	331
100. The Cycle is more relaxed. it gives me time to learn	4.00	1.11	331
98. I value having fewer teachers per course	3.95	1.16	326
108. Studying a Cycle improves my self−esteem	3.84	1.19	326
97. The teachers facilitate my study. they are colleagues	3.81	1.18	333
105. I have good classmates and that helps me	3.97	1.15	332
107. Studying a Cycle is useful	4.15	1.09	330
109. Studying a Cycle helps me with my family	3.73	1.22	329
96. I feel like part of the class and they pay attention to me	4.05	1.15	332
106. If I don’t understand something. they repeat it until I understand	4.04	1.13	330
95. The teachers of the cycles help me	3.86	1.16	331
Total	**3.90**	**1.15**	**335**
Factor 2: Empowerment and improvement of the situation (EE).			
102. I’m better in the Cycle than in the ESO	4.22	1.20	327
104. I come to class excited. it’s worth the effort	4.08	1.04	328
94. This is the best thing that has happened to me in regard to my education	3.80	1.26	329
103. Now I am able to take on my own projects	4.00	1.09	334
101. I come to class at ease. happy	3.95	1.13	328
110. My attitude has improved	4.03	1.17	328
111. I think I will pass the Cycle without problem	4.05	1.08	331
Total	**4.01**	**1.13**	**335**

**Table 7:** Differences in the degree of satisfaction by sectors.

Satisfaction towards BTP Cycles		Sum of the Squares	gl	Quadratic Mean	F	Sig.	ε^2^
External support and adaptation to the cycle (AP)	Inter−groups	4.580	3	1.527	2.636	0.050 *	0.023
	Intra−groups	191.658	331	0.579			
	Total	196.238	334				
Empowerment and improvement of situation (EE)	Inter−groups	2.085	3	0.695	1.032	0.379	0.009
	Intra−groups	222.975	331	0.674			
	Total	225.061	334				

* *p* < 0.05.

**Table 8:** Rotated configuration matrix. Student goals.

Items	Factors	
	1	2
113. I want to join the working world	**0.712**	−0.212
119. I want to be useful to society	**0.708**	0.171
115. I want them to be proud of me	**0.670**	0.227
114. I want to become professionally qualified	**0.666**	0.297
112. I would like to be able to do a Middle Grade Cycle	**0.583**	0.464
117. I want to get the ESO Diploma	**0.581**	0.126
118. I want to get a higher or university degree	0.036	**0.830**
116. I want to continue studying	0.207	**0.758**
**Explained variance**	32.79	21.35

**Table 9:** Student satisfaction with the BPT cycles.

	M	DT	*N*
Factor 1: Socio−professional and intermediate academic levels goals (MP).			
112. I would like to be able to do a Middle Grade Cycle	3.97	1.34	332
113. I want to join the working world	4.04	1.23	327
114. I want to become professionally qualified	4.19	1.03	327
115. I want them to be proud of me	4.29	1.12	331
117. I want to get the ESO Diploma	4.37	1.09	332
119. I want to be useful to society	4.18	1.10	332
**Total**	**4.17**	**1.15**	**332**

## Data Availability

The data presented in this study are available upon request from the corresponding author.
